# Supplementary material for: RiboTag RNA Sequencing Identifies Local Translation of HSP70 in Astrocyte Endfeet After Cerebral Ischemia
Source: Int J Mol Sci. 2025 Jan 1;26(1):309. doi: 10.3390/ijms26010309 (PMC11720067; doi:10.3390/ijms26010309)
Supplement: Supplementary file 1 [file ijms-26-00309-s001.zip › Supplemental Figure S1.pdf]

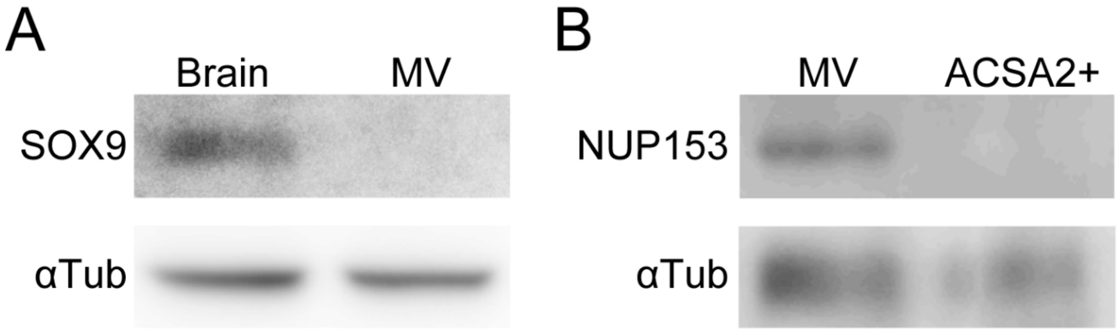

Supplemental Figure S1. Depletion of astrocyte somatic markers in post-ischemic microvessel isolates. (A) Protein lysates from the post-ischemic brain hemisphere or mechanically isolated microvessels were blotted for the astrocytic transcription factor SOX9 and the loading control α-tubulin. (B) Protein lysates from mechanically isolated post-ischemic microvessels or magnetically sorted ACSA2+ astrocyte endfeet were blotted for a nuclear pore complex (NPC) protein NUP153 and the loading control α-tubulin.
