# Supplementary material for: RiboTag RNA Sequencing Identifies Local Translation of HSP70 in Astrocyte Endfeet After Cerebral Ischemia
Source: Int J Mol Sci. 2025 Jan 1;26(1):309. doi: 10.3390/ijms26010309 (PMC11720067; doi:10.3390/ijms26010309)
Supplement: Supplementary file 1 [file ijms-26-00309-s001.zip › Supplemental Figure S2.pdf]

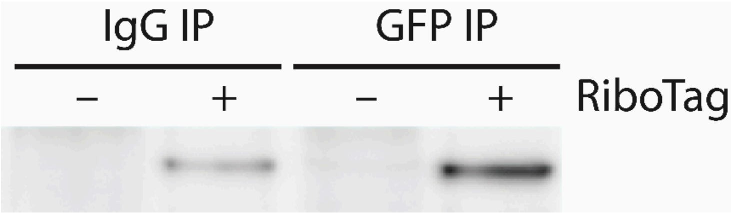

Supplemental Figure S2. Validation of a magnetic bead-conjugated anti-GFP antibody for immunoprecipitation of astrocytic EGFP-RPL10a. Representative immunoblot probed with anti-GFP antibody of brain lysates expressing *EGFP/Rpl10a* (RiboTag +; lanes 2 and 4) or littermate controls (RiboTag -; lanes 1 and 3) following magnetic immunoprecipitation using control IgG (IgG IP; lanes 1 and 2) or anti-GFP (GFP IP; lanes 3 and 4). –
