# Supplementary material for: RiboTag RNA Sequencing Identifies Local Translation of HSP70 in Astrocyte Endfeet After Cerebral Ischemia
Source: Int J Mol Sci. 2025 Jan 1;26(1):309. doi: 10.3390/ijms26010309 (PMC11720067; doi:10.3390/ijms26010309)
Supplement: Supplementary file 1 [file ijms-26-00309-s001.zip › Supplementary Tables S1 and S2.pdf]

Table S1. List of antibodies for immunoassays used in this study.

Antibodies	Manufacturer	Catalog Number
Immunohistochemistry		
Rabbit polyclonal anti-GFP	Cell Signaling Technology	2555S
Guinea pig polyclonal anti-S100B	Synaptic Systems	287004
Rat monoclonal anti-CD31 (MEC13.3)	BD Biosciences	550274
Rabbit polyclonal anti-AQP4	MilliporeSigma	ab3594
Mouse monoclonal anti-AQP4 (4/18)	Santa Cruz	sc-32739
Mouse monoclonal anti-HSP70 (3A3)	Santa Cruz	sc-32239
Goat anti-guinea pig IgG Alexa Fluor 488	ThermoFisher	A11073
Donkey anti-rat IgG Alexa Fluor 555	ThermoFisher	A78945
Donkey anti-mouse IgG Alexa Fluor 488	ThermoFisher	A21202
Donkey anti-rabbit IgG Alexa Fluor 555	ThermoFisher	A31572
Donkey anti-rabbit STAR RED	Abberior	STRED-1002
Immunoblotting		
Rabbit polyclonal anti-GFP	Cell Signaling Technology	2555S
Rabbit polyclonal anti-HSP70	Cell Signaling Technology	4872T
Rat monoclonal anti-HSC70 (1B5)	Santa Cruz	sc-59560
Rabbit polyclonal anti-GFAP	Dako	z0334
Rabbit monoclonal anti-SOX9	Cell Signaling Technology	82630
Rabbit monoclonal anti-NUP153	Cell Signaling Technology	36936
Mouse monoclonal anti-ubiquitin (P4D1)	Cell Signaling Technology	3936
Rabbit monoclonal anti-tubulin (11H10)	Cell Signaling Technology	2125
Goat anti-rat IgG, HRP conjugated	Cell Signaling Technology	7077S
Horse anti-mouse IgG, HRP conjugated	Cell Signaling Technology	7076S
Goat anti-rabbit IgG, HRP conjugated	Cell Signaling Technology	7074S

Table S2. List of qPCR primer sequences used in this study.

Gene (Accession No.)	Forward Sequence (5′→3′)	Reverse Sequence (5′→3′)
Aqp4 (NM_009700)	AGCCAGCATGAATCCAGCTCGA	TCATAAAGGGCACCTGCCAGCA
Gfap (NM_010277)	CACCTACAGGAAATTGCTGGAGG	CCACGATGTTCCTCTTGAGGTG
Pecam1 (NM_008816)	CCAAAGCCAGTAGCATCATGGTC	GGATGGTGAAGTTGGCTACAGG
Cldn5 (NM_013805)	TGACTGCCTTCCTGGACCACAA	CATACACCTTGCACTGCATGTGC
Pdgfrb (NM_008809)	GTGGTCCTTACCGTCATCTCTC	GTGGAGTCGTAAGGCAACTGCA
Acta2 (NM_007392)	TGCTGACAGAGGCACCACTGAA	CAGTTGTACGTCCAGAGGCATAG
Hspa1 (NM_010479, NM_010478)	ACAAGTCGGAGAACGTGCAGGA	GTTGTCCGAGTAGGTGGTGAAG
Ptx3 (NM_008987)	CGAAATAGACAATGGACTTCATCC	CATCTGCGAGTTCTCCAGCATG
Thbs1 (NM_011580)	GGTAGCTGGAAATGTGGTGCGT	GCACCGATGTTCTCCGTTGTGA
Itpkb (NM_001081175)	ACGCTACAACCAGATGGACGAC	ATGTCCTTCCGCAAGCTAGGCT
Nrarp (NM_025980)	CAGACAGCACTACACCAGTCAG	CCGAAAGCGGCGATGTGTAGC
Heyl (NM_013905)	CTGGAGAAAGCTGAGGTCTTGC	ACCTCAGTGAGGCATTCCCGAA
Sfrp5 (NM_018780)	GAGATGCTGCACTGCCACAAGT	TGCTCCATCTCACACTGGGCAC
